# Supplementary figures and images for: miR-195-5p as Regulator of γ-Catenin and Desmosome Junctions in Colorectal Cancer
Source: Int J Mol Sci. 2023 Dec 3;24(23):17084. doi: 10.3390/ijms242317084 (PMC10707010; doi:10.3390/ijms242317084)

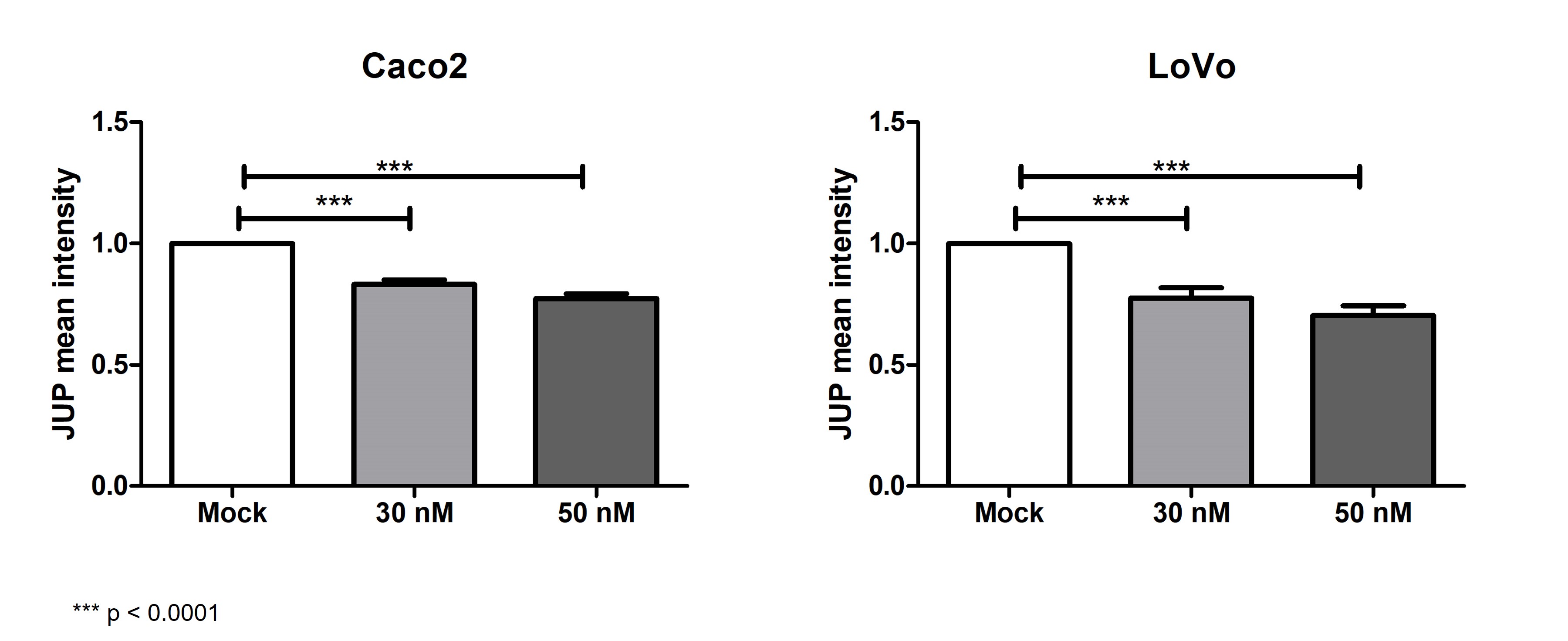

Supplement: Supplementary file 1 [file ijms-24-17084-s001.zip › Figure S1 Mean quantification of g-catenin staining intensity.jpg]
